# Identification of an Alternative rRNA Post-transcriptional Maturation of 26S rRNA in the Kingdom Fungi

**DOI:** 10.3389/fmicb.2018.00994

**Published:** 2018-05-25

**Authors:** Alfonso Navarro-Ródenas, Andrea Carra, Asunción Morte

**Affiliations:** ^1^Departamento Biología Vegetal (Botánica), Facultad de Biología, Universidad de Murcia, Murcia, Spain; ^2^Istituto per la Protezione Sostenibile delle Piante, Consiglio Nazionale delle Ricerche, Turin, Italy

**Keywords:** *Terfezia*, *Tirmania*, desert truffles, hidden gap, LSU rRNA, domain D7

## Abstract

Despite of the integrity of their RNA, some desert truffles present a non-canonical profile of rRNA where 3.3 kb is absent, 1.8 kb is clear and a band of 1.6 kb is observed. A similar rRNA profile was identified in organisms belonging to different life kingdoms, with the exception of the Kingdom Fungi, as a result of a split LSU rRNA called *hidden gap*. rRNA profiles of desert truffles were analyzed to verify the presence of the non-canonical profile. The RNA of desert truffles and yeast were blotted and hybridized with probes complementary to LSU extremes. RACE of LSU rRNA was carried out to determine the LSU rRNA breakage point. LSU rRNA of desert truffles presents a post-transcriptional cleavage of five nucleotides that generates a *hidden gap* located in domain D7. LSU splits into two molecules of 1.6 and 1.8 kb. Similar to other organisms, a UAAU tract, downstream of the breakage point, was identified. Phylogenetic comparison suggests that during fungi evolution mutations were introduced in the hypervariable D7 domain, resulting in a sequence that is specifically post-transcriptionally cleaved in some desert truffles.

## Introduction

The fungi called “desert truffles” or “turmas” comprise certain species of mycorrhizal fungi, most belonging to the order Pezizales (division Ascomycota), which produce hypogeous and edible fruit bodies. Their distribution is limited to arid and semi-arid regions, mainly in the Mediterranean Basin and the Middle East (Moreno et al., 2014; Zambonelli et al., 2014). The most appreciated genera for their edible ascocarps are *Terfezia* (Tul. & Tul.) Tul. & Tul., Picoa Vittad. 1831 and Tirmania Chatin 1892. Most of the species of *Terfezia, Picoa*, and *Tirmania* establish mycorrhizal symbiosis with plants of the family *Cistaceae*, mainly with perennial or annual species of the genus *Helianthemum* (Morte and Andrino, 2014). This symbiotic association plays a key role in the maintenance of Mediterranean shrubland and xerophytic grassland ecosystems, preventing erosion and desertification (Honrubia et al., 1992). In addition, *Terfezia claveryi* Chatin was the first desert truffle species to be cultivated (Morte et al., 2008, 2017). During recent years, this species has become an agricultural alternative in semi-arid areas due to its appreciated edible valuable product with low water requirements for cultivation (Morte et al., 2010).

Our most recent studies on desert truffles used molecular biology techniques, which often require the isolation of intact RNA (Navarro-Ródenas et al., 2012, 2013). As a routine, RNA evaluation is based on the integrity of the small (SSU) and large (LSU) subunits of ribosomal RNAs (rRNA) that must be visualized as sharp and clear ∼1.8 and ∼4.0 kb bands in a denaturing agarose gel or in a bioanalyzer. The canonical band profile in eukaryotic samples is a LSU rRNA band twice as intense as the SSU rRNA band. Nevertheless, in some organisms from a wide range of biological groups including bacteria, cyanobacteria, protozoa, insects, helminthes, fish and mammals, despite their RNA integrity, a non-canonical profile of rRNA bands has also been observed, where LSU is nearly or totally absent and a second or third band of different size may appear (Stevens and Pachler, 1972; Lava-Sanchez and Puppo, 1975; Pellegrini et al., 1977; Eckert et al., 1978; Leon et al., 1978; Leipoldt and Engel, 1983; Ware et al., 1985; Burgin et al., 1990; Mertz et al., 1991; van Keulen et al., 1991; Azpurua et al., 2013). In these organisms, an unusual post-transcriptional processing of LSU rRNA has been reported, which results in its breakage into two subunits called 28Sα and 28Sβ (Stevens and Pachler, 1972; Lava-Sanchez and Puppo, 1975; Pellegrini et al., 1977; Eckert et al., 1978; Leon et al., 1978; Ware et al., 1985; Burgin et al., 1990; Mertz et al., 1991; van Keulen et al., 1991; Azpurua et al., 2013). These subunits are non-covalently bound (Ishikawa and Newburgh, 1972; Ishikawa, 1977a,b; Winnebeck et al., 2010) and migrate near the 18S rRNA. The break point, called the “hidden break” (Ishikawa and Newburgh, 1972) was subsequently found not to be a single cleavage point, but the site of a double-cut event producing excised fragments of variable length in different species, and referred to as the “hidden gap” (Ishikawa, 1977a). In the case of naked mole-rat the presence of a cleaved 28S rRNA has been associated with increased translation fidelity which may contribute to the unusual longevity of this mammal (Azpurua et al., 2013).

The LSU rRNA hidden break has not been reported in fungi yet. However, when *T. claveryi* RNA is analyzed in a bioanalyzer after thermal denaturation, the non-canonical band profile is displayed, where LSU rRNA is almost missing and a typical SSU rRNA (1.8 kb) and a peak similar to bacterial 16S rRNA (1.6 kb) are clearly observed. We are perhaps faced with a unique event throughout the evolutionary history of the Kingdom Fungi. Our hypothesis is that the non-canonical rRNA band profile observed in some desert truffles is due to a specific post-transcriptional processing of LSU called “hidden gap,” even though it had not been observed in fungi before. To explain this pattern, the structure of *T. claveryi* LSU rRNA was studied using molecular techniques, with the main objective of uncovering the existence and mapping the position of a hidden gap in the LSU rRNA in fungi.

## Materials and Methods

### RNA Isolation and Microfluid Analyzer

Total RNA was isolated from, at least, three different samples of ascocarps of each of the desert truffles *T. claveryi, Terfezia arenaria* (Moris) Trappe and *Picoa lefebvrei* (Pat.) Maire, mycelium of *Tirmania nivea* (Desf.) Trappe or yeast using spin columns (RNeasy Plant Mini Kit, Qiagen) according to the manufacturer’s instructions. In the case of *T. claveryi*, the CTAB protocol (Chang et al., 1993) or the phenol and chloroform extraction (Kay et al., 1987) were also used for RNA extraction, both in ascocarps and mycelium.

For quality and integrity analysis, *T. claveryi, T. arenaria, T. nivea*, and *P. lefebvrei* RNAs were electrophoretically separated in agarose gel or with an Agilent 2100 Bioanalyzer, using a 2100 expert_Eukaryote total RNA Nano Chip. When RNA was heat denatured prior to separation, RNA was incubated at 95°C for 2 min.

### Northern Blots

RNA was electrophoresed in denaturing 1.2% (w/v) agarose gels prepared in double-distilled H_2_O and 1X MOPS buffer. Formaldehyde was added to a final concentration of 7% (vol/vol). Samples were denatured at 65°C for 10 min, placed on ice, and then loaded. Electrophoresis was carried out at ∼50 V for 6 h in 1X MOPS containing 1,5% formaldehyde as running buffer. Samples were blotted to positively charged Nylon membranes (Roche) in SSC 10X overnight. The membranes were hybridized with probes (Supplementary Figure S1) labeled with the PCR DIG labeling mix (Roche) using the primers listed in **Table 1**. Hybridizations were performed at 50°C in DIG Easy Hyb (Roche) overnight. Stringency washings were performed with 0.1X SSC- 0.1% (w/v) SDS, at 68°C for 30 min. Target RNA was detected according to the manufacturer’s protocol (Roche Diagnostics, Germany) using CSPD as chemiluminescent substrate.

**Table 1:** List of primers used.

Primer code	DNA sequence (5′–3′)	Sense	Use
26S_5′_for	GTGAAGCGGCAAAAGCTCAGAT	S	26S_5′-DIG-probe
26S_5′_rev	TTCCAACCCCAAGGCCTCTAAT	AS	26S_5′-DIG-probe
LR10R	GACCCTGTTGAGCTTGA	S	26S_3′-DIG-probe
LR12	GACTTAGAGGCGTTCAG	AS	26S_3′-DIG-probe
Adaptor	ACGCTACGTAACGGCATGACAGTG		26Sα_3′-RACE
Oligo-(dT)-adaptor	GCTGTCAACGATACGCTACGTAACGGC ATGACAGTGd(T^12^)VN		26Sα_3′-RACE
LR3R	GTCTTGAAACACGGACC	S	26Sα_3′-RACE
LR7R	TACTACCACCAAGATCT	S	26Sα_3′-RACE
LR9	AGAGCACTGGGCAGAAA	AS	26Sβ_5′-RACE
5′RACE Oligo-(dT)-adaptor	GCTGTCAACGATACGCTACGTAACGGCAT GACAGTG(T^17^)VN		26Sβ_5′-RACE
5′RACE_out_adaptor	TACGCTACGTAACGGCATGA		26Sβ_5′-RACE
5′RACE_adaptor	ACGCTACGTAACGGCATGACAGTG		26Sβ_5′-RACE
5′RACE_RT	TAGGGACAGTGGGAATCTCG	AS	26Sβ_5′-RACE
Ter26S_5′-RACE_Nest2	ATTCCCCCAGTCCGTACCAGTTCT	AS	26Sβ_5′-RACE
Ter26S_5′-RACE_Nest3	GGGTACGACCTGGCGTGAAAATTA	AS	26Sβ_5′-RACE

### RACE

RACE experiments were carried out as described by Scotto–Lavino et al. (2006) with minor modifications. For 5′ RACE 2 μg of DNase-treated RNA were denatured at 85°C for 5 min and reverse transcribed with Superscript IV (Invitrogen) in a 20 μl reaction at 55°C for 10 min using a *T. claveryi* 5′ RT specific primer. After digestion of residual RNA with 1 U of RNase H (Invitrogen), cDNA was purified with a silica column (Wizard^®^ SV Gel and PCR Clean-Up System, Promega) and poly(A) tailed with terminal transferase (Roche). Three rounds of PCR were performed, the first with primers 5′ RACE oligo dT adaptor, LR9 and 5′ RACE outer adaptor, the second with primers 5′ RACE adaptor and 26S 5′ RACEnest2 and the third with primers 5′ RACE adaptor and 26S 5′ RACEnest3. For 3′ RACE, 2 μg of DNase-treated RNA (Turbo DNA-free kit, Ambion) were polyadenylated with Poly(A) polymerase (Ambion) and reverse transcribed with an oligo-(dT)-adapter primer. First round and nested PCRs were performed with the primers shown in **Table 1** and Supplementary Figure S1. PCR products were cloned into pCR 2.1 TOPO vector (Invitrogen) and sequenced in forward and reverse orientation using universal primers (M13) recognizing vector sites encompassing the cloning site.

The nucleotide sequences of LSU rDNA were obtained from Joint Genome Institute (JGI^1^) for *T. boudieri* (Terbo2; JGI), *T. claveryi* (Tercla1; JGI), *Tuber melanosporum* (Tubme1; JGI), *Pyronema confluens* (Pyrco1; JGI) and *Morchella conica* (Morco; JGI), and *Saccharomyces cerevisae* (NR_132209.1) from NCBI. Moreover, we sequenced and deposited at NCBI the region encompassing the hidde gap of *T. arenaria* (MH160402), *Terfezia extremadurensis* (MH160403), *Terfezia fanfani* (MH160404), *T. nivea* (MH160405). All the sequences were aligned using the program ClustalO (Sievers et al., 2011).

## Results

### Electrophoretic Profiles of the Heat-Denatured and Native RNA Samples From *Terfezia* spp., *Tirmania nivea* and *Picoa lefebvrei*

**FIGURE 1:**
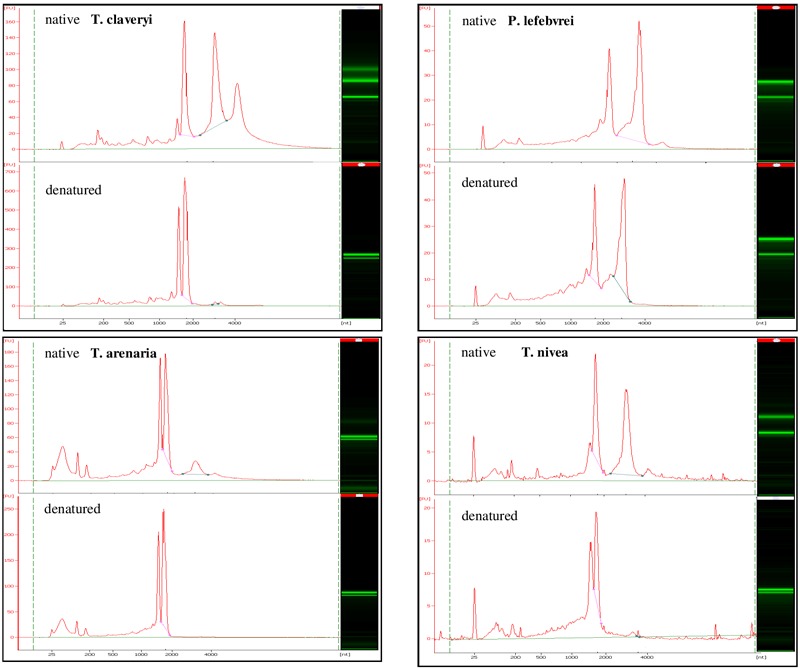
Bioanalyzer electrophoretic profiles of *T. claveryi, P. lefebvrei, T. arenaria* and *T. nivea* RNAs without heat-denaturation (native) and after heat-denaturation (denatured); nt, nucleotide length; FU, fluorescence unit.

The integrity of total RNA samples from *T. claveryi, T. arenaria, T. nivea*, and *P. lefebvrei* in heat-denatured or native conditions was assayed. For all the species, in native conditions, both the SSU and the LSU rRNA bands were identified (**Figure 1**). In addition, a peak similar of 1.6 kb was barely but clearly observed in *T. claveryi, T. arenaria*, and *T. nivea* (**Figure 1**). In *T. arenaria* the LSU peak seems smaller than in other species but this is the result of a partial denaturation during RNA extraction (Leaver, 1973). The third peak, higher than 4.0 kb and observed in all species under native condition, should be interpreted as an artifact. After heat denaturation, RNA samples from *P. lefebvrei* did not show significant differences in band profile, conversely, the presence of the LSU fragment was no longer observed in *T. claveryi, T. arenaria*, or *T. nivea* (**Figure 1**). On the other hand, the peak belonging to a fragment of approximately 1.8 kb, typical of the 18S rRNA, was still observed and the intensity of the 18S band had increased, as indicated by a greater FU (fluorescence unit) value in comparison with the FU value of the 18S rRNA peak for each non-heat-denatured sample (**Figure 1**). The presence of a second anomalous peak with an approximate size of 1.6 kb and intensity similar to the 18S peak was observed only in *T. nivea* and *Terfezia* species RNA (**Figure 1**). Low molecular weight RNA fractions, including a peak with an approximate size of 190 bp corresponding to 5.8S, were observed in both heat-denatured or non-heat-denatured conditions with similar intensity. Similar band profiles were observed in several ascocarps and with different RNA extraction protocols for all the species. For *T. claveryi*, the band profiles were also confirmed in RNA extracted from mycelium.

### *Terfezia* LSU Is Fragmented in Two Asymmetrical Subunits

**FIGURE 2:**
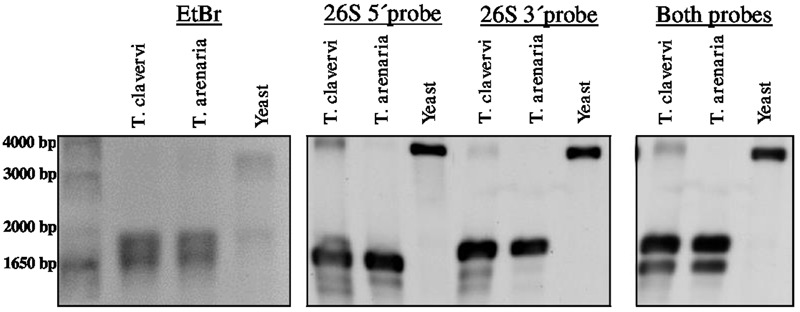
Denaturing formaldehyde-agarose gel electrophoresis of total RNA from *T. claveryi, T. arenaria* and yeast, stained with ethidium bromide. Northern blots of *T. claveryi, T. arenaria* and yeast rRNA probed with probes mapping to the 5′ and 3′ ends of LSU. Marker is 1 kb plus (Invitrogen).

Preliminary attempts to amplify different portions of the LSU rRNA by RT-PCR always failed when primers where designed encompassing a region of about 1 kb asymmetrically positioned within the 3′ half of the LSU rRNA molecule. On the basis of this observation and to confirm the presence of the hidden gap, two probes were designed, named 26S_5′ and 26S_3′, complementary to the 5′terminal and 3′terminal regions of *T. claveryi LSU rRNA*, respectively. They were used in Northern blot experiments with total RNA from *T. claveryi, T. arenaria* and yeast. In the case of yeast total RNA, both 26S_5′ and 26S_3′ probes detected the same RNA species of approximately 3.3 kb (**Figure 2**). However, in the case of *T. claveryi* and *T. arenaria* RNA, the 26S_5′ probe detected an RNA species of approximately 1.6 kb and the 26S_3′ probe detected another of approximately 1.8 kb (**Figure 2**). A very faint 3.3 kb band was also detected in *T. claveryi* RNA, suggesting that very low amounts of the uncut species were still present. When the blot hybridized with the 3′ probe was reprobed with the 5′ probe, no change in the 26S band of yeast rRNA was observed, but in both *Terfezia* species a distinct signal of about 1.6 kb was observed below the pre-existing 1.8 kb band (**Figure 2**). These results allowed us to conclude that: (i) the hidden gap maps within 1.6 kb from the 5′extreme and 1.8 kb from the 3′extreme of LSU, (ii) the 1.8 kb band, co-migrates with uncleaved 18S RNA and corresponds to the 3′ portion of LSU rRNA, (iii) the faint 3.3 kb band observed in *T. claveryi* corresponds to an uncut version of LSU rRNA and (iv) the absence of 28S rRNA is not due to a non-specific degradation.

### Hidden Gap Cleavage Points Are Conserved in *Terfezia* Species

**FIGURE 3:**
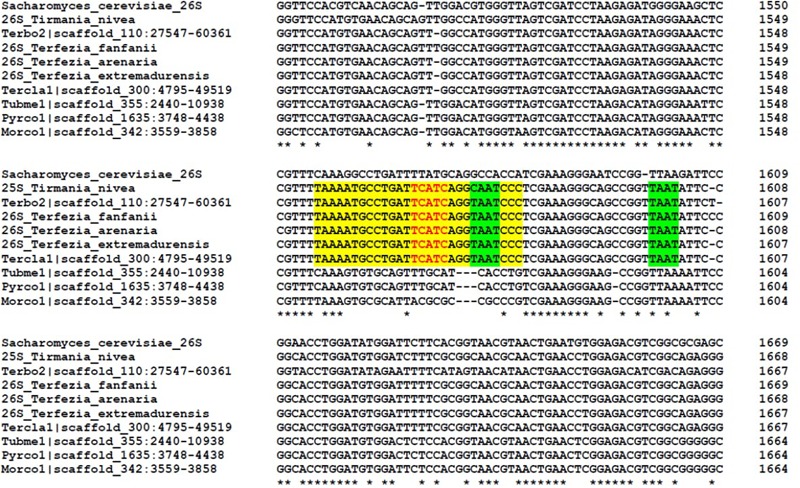
Alignment of the nucleotides encompassing the hidden gap of 26S rDNA sequences of *T. boudieri* (Terbo2; JGI), *T. claveryi* (Tercla1; JGI), *Tuber melanosporum* (Tubme1; JGI), *Pyronema confluens* (Pyrco1; JGI), *Morchella conica* (Morco; JGI), *T. arenaria* (MH160402), *T. extremadurensis* (MH160403), *T. fanfani* (MH160404), *T. nivea* (MH160405), and *Saccharomyces cerevisae* (NR_132209.1). Sequences were aligned using the Clustal Omega program. Asterisks indicate the nucleotides that are identical in all sequences analyzed. Red nucleotides indicate the hidden gap. Nucleotides shaded yellow indicate a region conserved in all hidden gap species but divergent in other species. Nucleotides shaded green indicate the possible UAAU tracts downstream of the 26S β segment (Macharia et al., 2015).

To determine the site and sequence of the *T. claveryi* 28S rRNA breakage point, primers to perform 5′ RACE and 3′ RACE were designed. Two cleavage sites, and between them a gap of five nucleotides, were identified after aligning with the *T. claveryi* LSU rDNA sequence (**Figure 3**). Different rDNA sequences of *Terfezia* spp. and *T. nivea*, which are known to present non-canonical rRNA profiles, were aligned with other Pezizomycetes fungi. All the tested *Terfezia* and *Tirmania* rDNA sequences are conserved in the five nucleotide gap, nine nucleotides upstream the gap and 10 nucleotides downstream the gap. In fact, these 24 nucleotides (shaded in yellow in **Figure 3**) seem to be a hypervariable region D7 within the LSU. This region D7 has been identified for being the closest downstream variable region to the conserved primer LR7 in the alignment. Four nucleotides downstream of the 26Sβ_5′extreme can be identified as a UAAU tract (**Figure 3**).

## Discussion

The LSU gene is organized into several highly conserved nuclei interrupted by 12 divergent domains, also called “expansion segments” (Clark et al., 1984) or “D domains” (Hassouna et al., 1984). Unlike nuclei, D domains show a high rate of insertions and deletions (Hassouna et al., 1984; Olsen and Woese, 1993). The fundamental importance of rRNA for the synthesis of proteins imposes evolutionary constriction on this type of structures. The insertion of sequences that alter the structure of the rRNA in the divergent domains would be negatively adaptive, unless they were accompanied by the removal of this inserts in the mature rRNA, similar to the excision of the ITS and ETS (internal and external transcribed spacer) (Melen et al., 1999). If the excision occurs in a region that does not need to be covalently continuous to be functional, the result can be a functional “fragmented” rRNA (Melen et al., 1999; Azpurua et al., 2013). In a wide range of biological groups, a double-cut event producing excised fragments of variable length, referred to as “hidden gap” (Ishikawa, 1977a), has been reported, resulting in a non-canonical profile of rRNA bands where 28S rRNA band is nearly or totally absent.

In this study, the lack of 28S rRNA after heat treatment in some desert truffles, is not due to unspecific degradation since the resulting fragments can be detected by Northern blot. Rather, it should be interpreted as a cleavage molecule with hydrogen bonds joining the 3′ and 5′ portions (28Sα and β, respectively). In this respect, the Northern blot of *T. claveryi* allowed also us to identify a very faint band, suggesting that very low amounts of the immature species were still present (Applebaum et al., 1966; Ishikawa and Newburgh, 1972) or that the hidden gap species of LSU is specific of some cell types (Melen et al., 1999; Azpurua et al., 2013), which consitute the ascocarp although the same post-transcriptional cleavage was also observed in mycelium.

This post-transcriptional maturation of *T. claveryi* LSU rRNA results in two molecules of 1.6 and 1.8 kb, corresponding to the 5′ and 3′ ends, which has not been previously observed in fungi even when it seems to be widespread in others kingdom, including bacteria, protozoa, worms, several arthropods, fish and rodents (Stevens and Pachler, 1972; Lava-Sanchez and Puppo, 1975; Pellegrini et al., 1977; Eckert et al., 1978; Leon et al., 1978; Leipoldt and Engel, 1983; Ware et al., 1985; Burgin et al., 1990; Mertz et al., 1991; van Keulen et al., 1991; Azpurua et al., 2013).

This breakage was located by RACE between 1,566 and 1,570 nucleotides from LSU 5′ extreme. The hidden gap region and the nucleotides surrounding it are located within the D7 domain (Hassouna et al., 1984). Although the size of the gap region differs among organisms, the gap sequence resided in the same region within the 28S rRNA, locating in the D7a expansion segment of eukaryotes (Hassouna et al., 1984; Rijk et al., 1994).

To date, the species where the hidden gap has been observed belong to *Terfezia* genus [*T. claveryi, T. arenaria* (**Figure 1**), *T. extremaduriensis, T. fanfani, T. boudieri* (data not shown)] and even to the closest genus *Tirmania* (**Figure 1**), but have not been reported in other Pezizomycetes such as *Picoa lefbvrei* (**Figure 1**), *Tuber melanosporum, Morchella conica*, or *Pyronema confluens*. During Pezizomycetes evolution, the introduction of sequences susceptible to cleaving to form a hidden gap must have appeared relatively late in their evolution, since, as far as we know, few genera share the hidden gap within the Pezizomycetes order.

The molecular mechanism behind the introduction of this hidden break remains unclear. Microinjecting *Sciara coprophila* rDNA into *Xenopus laevis* oocytes confirmed that during pre-28S rRNA fragmentation, specific processing machinery is not required. Instead, oocytes may provide the required accessory factors, suggesting that the gap processing mechanism is served by an evolutionarily conserved apparatus (Basile-Borgia et al., 2005). Alternatively, these results may suggest that processing in some lineages is an autocatalytic property of the rRNA (Basile-Borgia et al., 2005). Nishimura et al. (2010) showed that RH39 is a DEAD box protein involved in post-maturation processing of the hidden gap in chloroplast 23S rRNA and, recently, RH50 has been identified as another plastid 23S rRNA maturation factor for correct 23S rRNA cleavage (Paieri et al., 2017). The cleavage site has been correlated with a UAAU tract downstream of the β segment (Fujiwara and Ishikawa, 1986; Ogino et al., 1990; Sun et al., 2012; Macharia et al., 2015). As well as other organisms, *T. claveryi* and *T. boudieri* present the UAAU tract downstream of the 26Sβ rRNA.

Regarding the possible biological role of this post-transcriptional maturation, several interpretations have been proposed. One theory proposes a compensatory protein translation rate of hidden gap ribosomes since they show reduced efficiency for protein synthesis *in vitro* within tetraploid Cyprinid species (Leipoldt and Engel, 1983). The second interpretation is based in the substantially higher translation fidelity of naked mole rat cells with respect to mouse cells. Naked mole rat is well-adapted to extreme environments and has an unusually long life span, which has been correlated with higher translation fidelity of its ribosomes (Azpurua et al., 2013). Additionally, the chloroplast ribosomes of an *Arabidopsis thaliana* mutant, which exhibit defective chloroplast 23S rRNA fragmentation, have a drastically reduced level of ribulose-1,5-bisphosphate carboxylase/oxygenase and of other chloroplast-encoded photosynthetic proteins (Nishimura et al., 2010). The third interpretation proposes a ribosomal inactivating control mechanism in response to stress conditions by involving the splitting of 60S subunits containing 28S rRNA with a central hidden break (Nomura et al., 2016). *Terfezia* species, which are also adapted to extreme environments, display high resistance to drought and high temperatures (Navarro-Ródenas et al., 2011, 2012, 2013; Honrubia et al., 2014; Zambonelli et al., 2014). Whether *Terfezia* 28S rRNA influences translation efficiency and what impact this can have on its biological cycle is still unknown and deserves further research.

## Author Contributions

AN-R, AC, and AM conceived and designed research. AN-R and AC completed the experiments and analyzed the data. AN-R wrote the manuscript. AC and AM reviewed the manuscript. AM provided financial support.

## Conflict of Interest Statement

The authors declare that the research was conducted in the absence of any commercial or financial relationships that could be construed as a potential conflict of interest. The handling Editor declared a shared affiliation, though no other collaboration, with one of the authors AC.

## Footnotes

1 https://genome.jgi.doe.gov
