# Supplementary material for: Identification of an Alternative rRNA Post-transcriptional Maturation of 26S rRNA in the Kingdom Fungi
Source: Front Microbiol. 2018 May 25;9:994. doi: 10.3389/fmicb.2018.00994 (PMC5981135; doi:10.3389/fmicb.2018.00994)
Supplement: Supplementary file 1 [file Image_1.pdf]

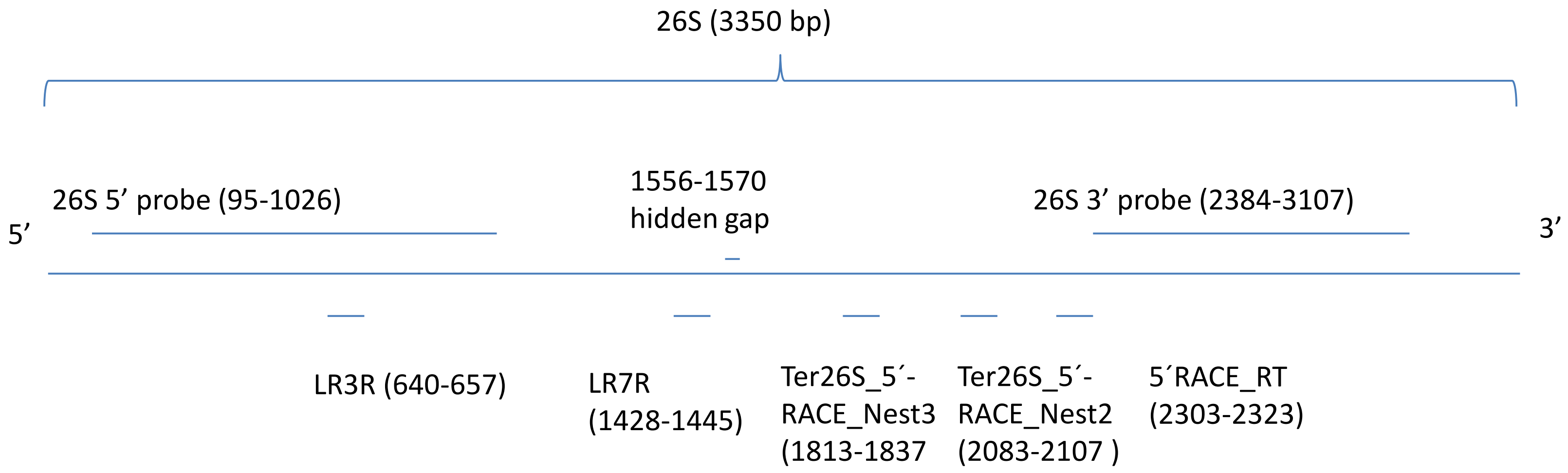

Supplementary Fig. S1. Schematic representation of *Terfezia claveryi* 26S RNA with probes used for Northern blot hybridizations and primers used in 3’ and 5’ RACE to map the position of the hidden gap. Numbers in parenthesis mean the nucleotide positions.
